# Liquid Deposition Modeling of Biobased Epoxy Composites: Natural Fillers as Rheology Modifiers and Reinforcements

**DOI:** 10.1021/acsomega.5c10820

**Published:** 2026-02-06

**Authors:** Edoardo Albertini, Christos Fragkogiannis, Lucia Tsantilis, Rossella Arrigo, Alessandra Vitale, Roberta Bongiovanni, Sara Dalle Vacche

**Affiliations:** 1 19032 Politecnico di Torino, Corso Duca degli Abruzzi 24, Turin 10129, Italy; 2 19032 Politecnico di Torino, Viale Teresa Michel, Alessandria 15121, Italy

## Abstract

In this work, aimed at developing biomass-based composite pastes for liquid deposition modeling (LDM) 3D printing, we investigated the tuning of the rheological properties of a cardanol-based epoxy resin through the incorporation of various fillers: microcrystalline cellulose (MCC), microfibrillated cellulose (MFC), and nanoclay (MMT). The thermal cross-linking of the amine-cured composite pastes was monitored by ATR-FTIR and DSC analyses, confirming complete conversion of epoxy functionalities. The rheological behavior of the uncured composites was studied in view of LDM 3D printing. Viscosity data were fitted with the Herschel–Bulkley model to determine yield stress (τ_0_), consistency index (*K*), and flow behavior index (*n*). Shear-thinning behavior with solid-like to liquid-like transition at relatively low strain (0.5–5%) was induced by the addition of fillers, with adequate structural recovery. MFC proved to be the most effective rheological and mechanical property enhancer but could not be used alone due to curing-induced shrinkage at high loadings. Partial substitution of MCC with MFC, instead, drastically increased viscosity and reinforced shear thinning while retaining solid-like behavior at rest and yielded the highest tensile mechanical properties. In contrast, partial substitution of MCC with MMT slightly improved the tensile properties without significantly changing the rheology. Overall, increasing the filler content improved the mechanical properties of the composites to an extent that depended on the type and amount of filler. An optimized formulation containing 22 vol % of MCC and 1 vol % of MFC showed promising properties for LDM 3D printing, exhibiting proper extrusion (τ_0_ = 281.54 Pa, *K* = 855.43 Pa·s^*n*^, and *n* = 0.57), good shape fidelity, and, after curing, tensile modulus and strength equal to 5.34 and 1.31 MPa, respectively.

## Graphical abstract

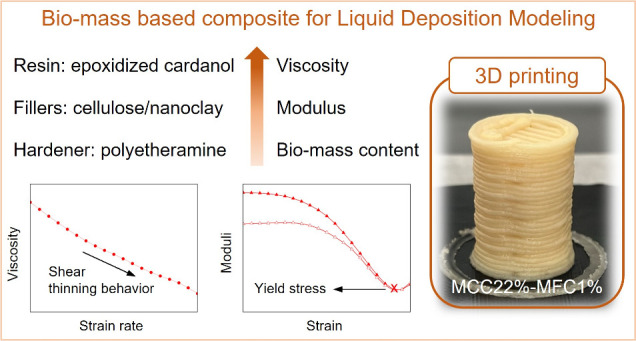

## Introduction

Polymer additive manufacturing (AM) has seen significant growth across a wide range of application areas in recent years.^1^ Among the various 3D printing techniques, liquid deposition modeling (LDM) demonstrates great flexibility and adaptability to a wide range of materials.^2^ In LDM, similarly to direct ink writing (DIW), highly viscous slurries or pastes with tailored rheological properties are extruded at ambient temperature through cold extrusion processes.^3^ For printability and shape retention, the slurries need to possess a shear-thinning behavior with a yield stress and a sufficient storage modulus at rest to support the deposition of superposing layers.^4^ Solidification is carried out postprinting with different mechanisms including drying, physical gelation, or cross-linking. For these characteristics, LDM has particular potential for shaping thermoset composite materials.^5^

As the technology advances, there is a growing interest toward replacing conventional petroleum-derived materials used in additive manufacturing with more sustainable and environmentally friendly alternatives, such as biobased, biodegradable, or recyclable polymers.^6^ Plant-derived oils, including, e.g., soybean and linseed oils, and particularly nonedible ones, such as tung, castor, or jatropha oils, provide a suitable platform for thermoset biobased materials.^7,8^ Cashew nutshell liquid (CNSL) represents a sustainable and renewable feedstock that is rich in phenolic constituents. Among these, cardanol is a versatile and valuable synthon that serves as a viable biobased alternative to petroleum-derived phenols in polymer production.^9–11^ Numerous epoxy resins have been successfully synthesized from cardanol, some of which are already commercialized.^12^ Nevertheless, being typically Newtonian fluids, they do not meet the rheological requirements for LDM 3D printing.

Micro- and nanosized cellulose fibers and clays are natural fillers known to impart shear-thinning properties to epoxy resins,^13^ making them of interest as rheology modifiers for LDM pastes. Furthermore, they act as reinforcements, increasing the mechanical properties of the cured composite materials.^14–16^

Cellulose, the most abundant biopolymer in nature, is widely used in the form of fibers, ranging from macro- to nanoscale, for the preparation of biobased composites.^14,17^ The physical and mechanical properties of cellulose fibers are significantly influenced by their morphologies and crystallinity. Microcrystalline cellulose (MCC) is obtained from purified cellulose by hydrolysis of cellulosic fibers, yielding micron-sized particles with high crystallinity, and is commercially available as dry powders or colloidal suspensions under different brand names.^18^ In previous studies of our group, MCC proved efficient in tuning the rheology of biobased poly­(furfuryl alcohol) and epoxy/carboxylic acid-based slurries for LDM printing.^19,20^ For the latter, however, defects appeared in the printed pieces after curing, which was performed at high temperature (200 °C), highlighting the need to increase the stiffness at rest preventing collapsing of the lower layers.^20^

Microfibrillated cellulose (MFC), consisting of long and flexible microfibrils with diameters ranging from 10 to 100 nm, is produced by mechanical refining and high-pressure homogenization, which use high shear forces to promote the separation of cellulose fibrils.^21^ MFC suspensions exhibit a pronounced shear thinning behavior.^22^ MFC has been proposed as rheology modifier for several products, including water-based paints, adhesives, consumer products, and crop-protecting formulations.^23^ Being only available as aqueous suspensions with low solid content, however, the use of MFC as rheology modifier in 3D printing formulations resulted in high shrinkage due to the evaporation of water during the solidification step.^24,25^

Clay is widely used in LDM, mostly in the form of aqueous pastes, but also combined with natural fibers and polymer binders such as alginate, cellulose, or starch.^26^ Organically modified nanoclay platelets were employed as rheology modifiers for formulating inks suitable for LDM 3D printing based on bisphenol A epoxy resins. Their incorporation increased viscosity and promoted desirable shear-thinning behavior, either when used alone or in combination with carbon microfibers and/or carbide whiskers.^27^ In epoxy/wood pulp composite inks, organophilic nanoclay improved flowability and shape fidelity of the printed pieces despite slightly reducing shear thinning and yield stress compared to inks containing the same amount of wood pulp.^28^

However, studies investigating the effect of natural fillers of different sizes, alone or combined, on the rheological and viscoelastic properties of biobased epoxy resins with the aim of developing sustainable slurries suitable for LDM 3D printing are still scarce. To enlarge the range of biobased materials suitable for polymeric LDM additive manufacturing, in this work, we explored formulations based on a commercial cardanol-derived epoxy resin and natural fillers, i.e., microcrystalline cellulose (in the micron size range) alone and combined with microfibrillated cellulose (submicron size) or clay (nano size). To enhance the dispersion of the hydrophilic fillers in the hydrophobic epoxy resin, we selected as hardener Jeffamine ED 900, a polyetheramine in which primary amino groups are attached to the end of a poly­(ethylene glycol) backbone, making the molecule compatible with hydrophilic compounds. The rheological behavior of the uncured slurries, fundamental for LDM printing, was thoroughly characterized, analyzing the effect of different combinations and volume fractions of fillers. Then, we studied the curing of the slurries and assessed the morphology and physical and mechanical properties of the cured composites. Interactions and synergistic effects among the fillers were highlighted. Finally, we evaluated the printability via LDM additive manufacturing of composite slurries selected based on the rheological and mechanical characterization, obtaining, with the optimal composite formulation, printed pieces with good shape fidelity before and after curing.

## Experimental Section

### Materials

The cardanol-based epoxy resin NC-514S, having an epoxide equivalent weight (EEW) of 426–438 g/eq and thus and average functionality close to 1.36, was supplied by Cardolite Specialty Chemicals Europe NV (Belgium). *O*,*O*′-Bis­(2-aminopropyl) polypropylene glycol-*block*-polyethylene glycol-*block*-polypropylene glycol 1800 (Sigma-Aldrich, US), known under the commercial name Jeffamine ED 900, was used as hardener: it is a water-soluble aliphatic polyether diamine with an amine hydrogen equivalent weight (AHEW) of 227 g/eq. The chemical structures of the epoxy resin and the amine are reported in Figure S1 in the Supporting Information. Three commercial cellulosic fillers were used: microcrystalline cellulose in the form of dry powder, Technocel FM8 (MCC), supplied by CFF GmbH & Co. KG (Germany), and two microfibrillated cellulose aqueous suspensions, Celova M150R-P, a paste with 20 wt % MFC content (MFC_20_), and Celova M250R-P, a paste with 11 wt % MFC content (MFC_11_), both kindly supplied by Weidmann (Switzerland). According to the datasheet and as confirmed by literature data,^19^ the particle diameter of MCC is between 6 and 12 μm. The certificates of analysis provided by the supplier declared a particle length of 9–10 μm and surface areas of 178 m^2^ g^–1^ for MFC_20_ and 250 m^2^/g for MFC_11_. The clay filler was a natural montmorillonite (MMT), Cloisite Na^+^ by BYK Additives (Germany), having according to the datasheet, a density of 2.86 g cm^–1^ and dry particle size of less than 25 μm (d_50_). All products were used as received.

### Preparation of Thermoset Resins and Composite Slurries

Curable resins were prepared by mixing NC-514S epoxidized cardanol and Jeffamine ED 900 hardener with stoichiometric ratio of epoxy groups to amine hydrogens (E/AH = 1) and with slight hardener excess (E/AH = 0.8), stirring by hand at room temperature until homogeneous mixtures were obtained. Given the mass of NC-514S (*m*_E_), the mass of hardener (*m*_A_) to be used was calculated using the EEW and AHEW declared in the certificates of analysis, with eq 1.

$$m_{A}=\frac{m_{E}}{E/\mathrm{AH}}\cdot \frac{\mathrm{AHEW}}{\mathrm{EEW}}$$ 1

As detailed in Supporting Information (in the section Selection of the Resin Formulation), despite slightly lower reactivity (Figure S2), the resin with E/AH = 1 showed better overall performance; thus, it was selected for the preparation of composites.

Composites with only MCC as filler were prepared with filler volume fractions ranging from 0.07 to 0.31, and composites with only MFC were prepared with filler volume fractions equal to 0.07 and 0.23. Composites were also prepared with hybrid fillers, i.e., combining MCC with either MFC or MMT. The prepared composites with MCC were taken as a reference, and part of the MCC was substituted with an equivalent volume of MFC or MMT. The volume of MFC is always intended as volume of dry microfibrils, calculated from the cellulose contents of the MFC pastes. The fillers’ volume fractions in the composites are detailed in Table 1.

**Table 1:** Codes Identify the Composites and Volume Fractions of Fillers

composite	total (*V*_f_)	MCC (*V*_MCC_)	MFC_11_ (*V*_MFC11_)	MFC_20_ (*V*_MFC20_)	MMT (*V*_MMT_)
hand mixing					
MCC7	0.07	0.07			
MFC_11_7	0.07		0.07		
MFC_20_7	0.07			0.07	
MCC23	0.23	0.23			
MFC_11_23	0.23		0.23		
MFC_20_23	0.23			0.23	
MCC29	0.29	0.29			
					
planetary centrifugal mixing					
MCC23	0.23	0.23			
MCC22-MFC_20_1	0.23	0.22		0.01	
MCC29	0.29	0.29			
MCC28-MFC_20_1	0.29	0.28		0.01	
MCC28-MMT1	0.29	0.28			0.01
MCC31	0.31	0.31			
MCC29-MFC_20_2	0.31	0.29		0.02	
MCC29-MMT2	0.31	0.29			0.02

Two mixing methods were used: hand mixing and planetary centrifugal mixing. For hand mixing, the necessary amount of MCC or MFC paste was first gently crushed with pestle and mortar to break macroscopic aggregates. Subsequently, the hydrophilic amine hardener was added to the mortar, and thorough mixing was performed until a homogeneous mixture was achieved. Finally, epoxidized cardanol was added to the mixture, continuing to stir the slurry in the mortar until it became homogeneous. For planetary centrifugal mixing, a Thinky Mixer ARE-250 CE was used. For composites containing only MCC or MFC, all compounds were placed in the mixer in a polypropylene container and the mixing and defoaming program were started. For composites with *V*_MCC_ up to 0.23, a revolution speed of 1000 rpm was applied for 2 min in the mixing mode, and a revolution speed of 400 rpm was applied for 1 min in the defoaming mode. For composites with higher filler volume fractions of MCC and for the composites with MFC, the mixing mode revolution speed was increased to 2000 rpm and was still applied for 2 min. For hybrid composites, the slurries were prepared by planetary centrifugal mixing in two steps. First, the epoxy resin, the hardener, and the desired amount of either clay (MMT) or microfibrillated cellulose (MFC) paste were placed in the PP container and mixed with a mixing mode revolution speed at 2000 rpm for 2 min and a defoaming mode revolution speed at 400 rpm for 1 min. Then, the necessary amount of MCC was added to the mixture and mixed again by using the same settings.

### Thermal Curing of Resin and Composites

Before curing, the nonfilled thermosetting resin and the composite slurry MCC7 were degassed in vacuum for 30 min prior to casting in silicon open molds and then further degassed in vacuum for additional 30 min. All other slurries could not be degassed in a vacuum because of their very high viscosity and were directly placed in the molds with a spatula.

Resin and composites underwent a thermal curing process in a convection oven (Mod 2100. High Performance, F.lli Galli, Italy) consisting of sequential isothermal stages at increasing temperatures: 40 °C for 16 h, 80 °C for 2 h, 120 °C for 2 h, and 140 °C for 2 h. For the planetary centrifugally mixed MCC29 composites only, two shorter alternative thermal curing cycles were also investigated by skipping the lower temperature steps, but samples with worse morphology, presenting larger and more elongated voids, were obtained (optical micrographs in Figure S3 in the Supporting Information). Thus, the complete curing cycle was retained for all of the composites.

### Characterization

Rheological measurements were carried out by making use of Dynamic Shear Rheometers (MCR302 and MCR301 models from Anton Paar) equipped with parallel plates and coaxial cylinder sensor systems. The selected parallel plate devices were a PP08 (used for sample MCC28-MFC_20_1), with an 8 mm diameter and a 2 mm gap, and a PP25 (used for all the other samples), with a 25 mm diameter and 1 mm gap between upper and lower plates. The coaxial cylinder system was a CC17 apparatus, characterized by a measuring gap between external cup and internal measuring bob of 0.7 mm. Tests were conducted in both oscillatory and continuous mode at a constant temperature of 25 °C. In oscillatory mode, strain amplitude sweeps were carried out at a frequency of 1 Hz covering four decades of strain amplitudes comprised between 0.01% and 100%. Tests in continuous mode were flow and stepwise tests. In flow tests, the dynamic viscosity of materials was measured as a function of the shear rate, imposing a first down-ramp cycle immediately followed by a second up-ramp cycle of shear rate. Although shear rates comprised between 0.01 s^–1^ and 10 s^–1^ were investigated, for some of the composites, this range was restricted to avoid edge instability at high shear rates or excessive noise in data acquisition at low shear rates. Stepwise tests were conducted to monitor dynamic viscosity over time at two levels of shear rate. The upper and successive lower level of constant shear rates were applied twice, imposing a duration of each interval of constant shear rate equal to 50 s. A minimum of two replicates were run for each type of test.

Fourier transform infrared (FTIR) analysis was performed with a Nicolet iS50 spectrometer (Thermo Fisher Scientific Inc., Waltham, MA, US). The nonfilled resin was spread on a silicon wafer with a 10 μm wire wound bar, and after each isothermal step of the curing cycle, it was analyzed in transmission mode, in the 400–4000 cm^–1^ range, with 32 scans and a resolution of 4 cm^–1^. The same instrument fitted with an ATR-Smart Orbit accessory with a diamond crystal was used to follow the curing of thick specimens, both of nonfilled resins and composites, by attenuated total reflectance Fourier transform infrared (ATR FTIR) spectroscopy analyses, acquiring the spectra in the 525–4000 cm^–1^ range, with 32 scans per spectrum and a resolution of 4 cm^–1^. The degree of conversion of the epoxide was calculated with eq 2:

$$\alpha =1-\frac{A_{t}/A_{t}^{\mathrm{ref}}}{A_{t=0}/A_{t=0}^{\mathrm{ref}}}$$ 2

where *A* and *A* ^ref^ are the absorbances, taken as the areas of the corresponding peaks, of the signal of interest and of an internal reference signal corresponding to a bond that does not change during the reaction. The peak of interest was centered at 910 cm^–1^ (epoxy C–O bond). Considering as internal reference either the band of the aliphatic C–H bonds (3100–2700 cm^–1^) or the band of the aromatic ring C=C stretching (centered at 1580 cm^–1^), the calculated conversions were similar; the reported values are calculated using the latter.

The insoluble fraction of the cured resin and composites was assessed by measuring their mass before and after immersion in acetone or toluene, solvents that can completely dissolve the uncured resin. Samples were cut into small pieces to maximize the surface exposed to the solvent, wrapped in a fine metallic mesh, and immersed in the solvent for 24 h; then, they were extracted, and the residual solvent was evaporated at room temperature for 24 h followed by drying at 80 °C until no change in mass was detected.

The water uptake test was performed to check the amount of water absorbed by cross-linked samples. Samples of about 300 mg were weighed and soaked in 20 mL of distilled water. After 1 week, they were extracted from water, gently wiped, and weighed again. The water uptake was calculated as the percentage difference between the final and the initial mass.

Dynamic scanning calorimetry was performed with a DSC Q20 (TA Instruments, Div di Waters S.p.A., Italy) in closed aluminum pans, with a nitrogen flux of 50 mL min^–1^, a thermal cycle with two heating and one cooling step, in the temperature range from −70 to 180 °C, and heating/cooling rate of 10 °C min^–1^, with 3 min isothermal steps between each heating/cooling step. The glass transition temperatures were evaluated at the inflection points of the heat flow curves.

Thermogravimetric analysis was carried out under N_2_ flux from 50 to 800 °C at 10 °C/min on a Discovery TGA (TA Instruments, Div di Waters S.p.A., Italy).

Optical microscopy was performed in reflection mode with an Olympus BX53 M microscope (Olympus Italia S.R.L., Italy) equipped with a digital camera.

Freeze-fractured cross sections of the cured composites were observed with a Zeiss Supra Field Emission Scanning Electron Microscope (FESEM), with an aperture of 30 μm and a voltage of 3 kV. The samples were coated with a thin layer of platinum to prevent charging.

The mechanical properties of the cured resins and composites were evaluated in tensile configuration with an Instron universal mechanical testing machine equipped with Instron Series 2710-11x Screw Action Grips (Illinois Tool Work Inc., USA) and a load cell of 500 N, using a constant crosshead speed of 50 mm min^–1^. The tensile modulus was calculated from the slope of the linear part of the stress–strain curves. At least five dog bone-shaped specimens (ASTM D638-22, type IV) were tested for each type of material, and the average values and standard deviations were calculated for the modulus, tensile strength, and elongation at break.

### 3D Printing by LDM

The composite slurries with the highest cellulose weight fractions were 3D printed using a commercial benchtop printer, Sidewinder X1 (Artillery, HK) originally designed for FDM (Fused Deposition Modeling), and properly customized in house for LDM printing: the printer was fitted with a screw LDM clay extruder (WASP, Italy) having a nozzle diameter of 1 mm, and the slurry was fed to the extruder by a syringe kept under 2 bar pressure. The software Simplify3D was used as the slicer. After the flow calibration of the screw extruder, objects were printed with the layer height set at 1 mm, a printing speed of 500 mm/min, and 45% infill density. After printing, the objects were thermally cured using the same thermal process applied for the molded specimens, i.e., 40 °C for 16 h, 80 °C for 2 h, 120 °C for 2 h, and 140 °C for 2 h.

## Results and Discussion

Composite slurries with the filler volume fractions summarized in Table 1 were prepared by either hand mixing or planetary centrifugal mixing. Hand mixing proved suitable to prepare slurries with *V*_MCC_ up to 0.29; for higher volume fractions, it did not yield homogeneous mixtures. Slurries with *V*_MFC_ equal to 0.07 and 0.23 were prepared by hand mixing with both the MFC_11_ and MFC_20_ pastes. Planetary centrifugal mixing was then used for preparing slurries with *V*_MCC_ up to 0.31 and *V*_MFC_ equal to 0.23 (only using the most concentrated paste MFC_20_), and all the slurries with hybrid fillers, obtaining in all cases homogeneous materials.

### Rheological Characterization of Uncured Resin and Composite Slurries

Rheological measurements in oscillatory mode were performed on the hand-mixed and planetary centrifugally mixed composite slurries to monitor the evolution of the storage modulus (*G*′) and the loss modulus (*G*″) as a function of the shear strain. These tests clearly highlighted the influence of filler type, volume fraction, and mixing mode on the rheological behavior of the composites.

Strain amplitude sweeps allowed the identification of a linear viscoelastic domain followed by a nonlinear region, with a linear viscoelastic threshold that was found to be material dependent. Referring to the measurements performed on manually mixed slurries containing 23 vol % MFC (obtained using the two MFC suspensions at different concentrations) and 29 vol % MCC (Figure 1), it was found that the former showed a wider linear viscoelastic domain than the latter. Despite the lower filler volume fraction, the transition from a predominantly solid-like behavior to a predominantly liquid-like behavior occurred at higher strain levels for composites with 23 vol % MFC when compared to the ones containing 29 vol % MCC, as evident from the cross points between *G*′ and *G*″. It can also be observed that water introduced with MFC decreased the storage and loss moduli of the slurries, as with the same amount of filler, the moduli obtained with MFC_20_ (i.e., with the more concentrated paste) were higher than those obtained with MFC_11_.

**Figure 1:**
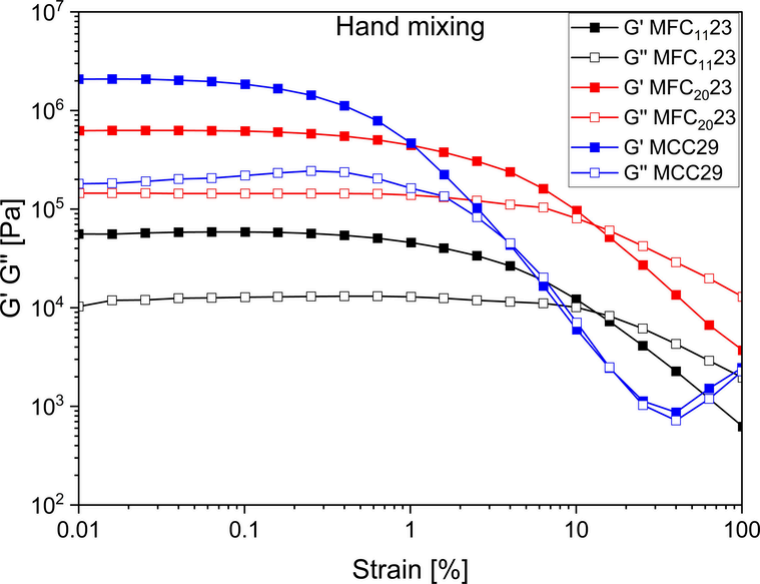
Strain amplitude sweeps of hand-mixed composites with MCC (29 vol %) and MFC (23 vol %).

Comparing planetary centrifugally mixed composites (Figure 2I) with the same filler type added at different volume fractions, it can be observed that higher volume fractions generally induced an increase in moduli and a shift of the *G*′ and *G*″ cross point to higher strains, to an extent that was found to be strictly dependent on the type of filler.

**Figure 2:**
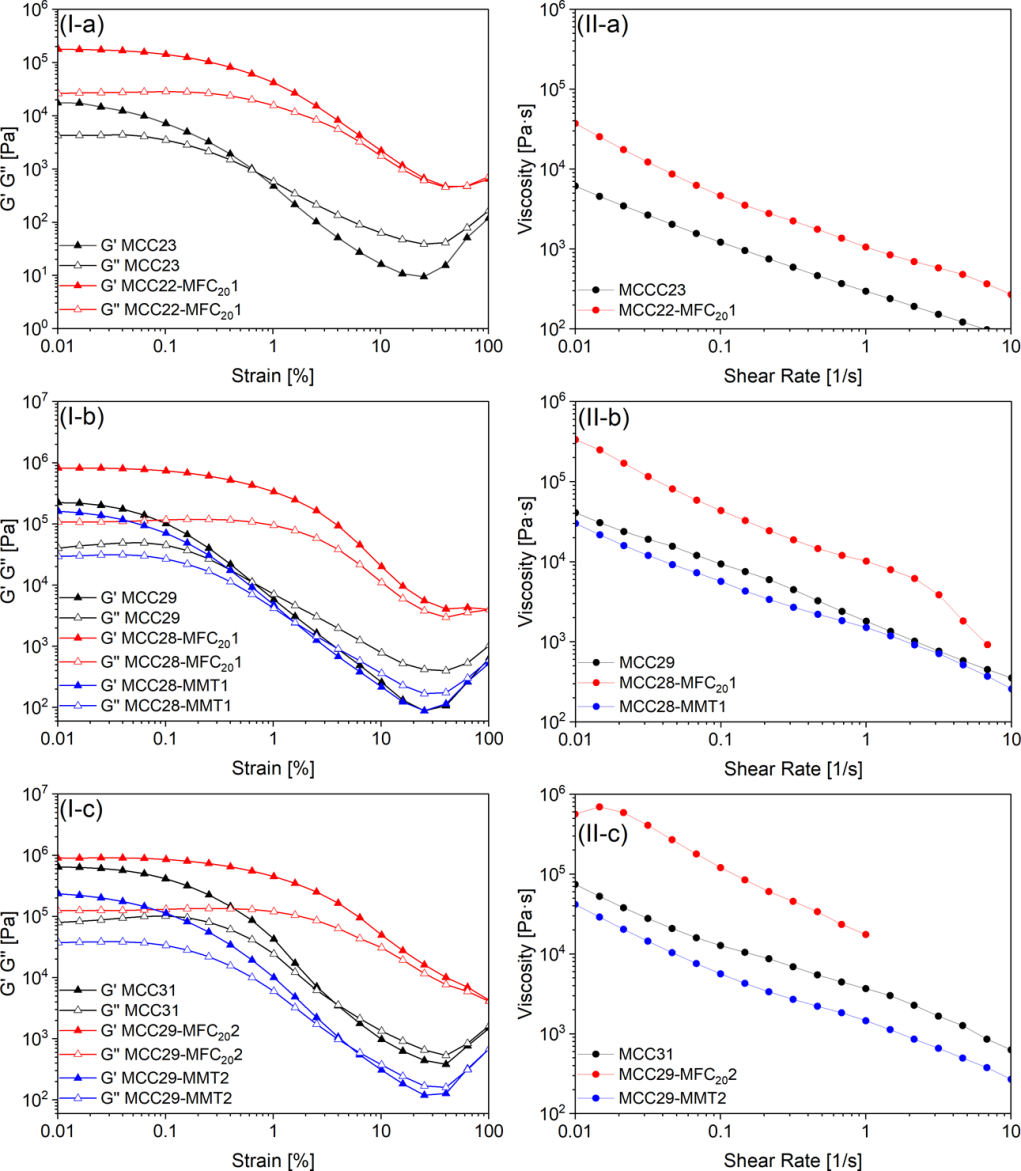
(I) Amplitude sweep curves and (II) flow curves of the composite slurries mixed by planetary centrifugal mixing: (a) pastes with 23 vol % of filler, (b) pastes with 29 vol % of filler, and (c) pastes with 31 vol % of filler.

Moreover, from the analysis of hybrid composites with either MFC or MMT containing equal volume fractions of filler, it can be claimed that MFC is more effective than MMT in affecting the rheological response of the base MCC composites. While MFC highly increases the loss and storage moduli of the slurries with respect to the same volume fractions of MCC, replacement of MCC with MMT results in even a slight reduction of the moduli. Furthermore, in MFC hybrid composites the linear viscoelastic domain is widened and crossing of *G*′ and *G*″ is shifted to higher strain amplitudes or is even not visible in the inspected range.

Rheological measurements in continuous mode were performed on planetary centrifugally mixed composites; the uncured resin was also characterized as a reference. Flow curves are shown in Figure 2II. Flow tests performed on the resin revealed a negligible shear-thinning response, with dynamic viscosity values ranging from about 2.1 Pa·s to 2.4 Pa·s at shear rates varying from from 0.01 to 100 s^–1^. Conversely to the resin, all composites showed a marked shear-thinning behavior in the range of investigated shear rates with measured values of dynamic viscosity that span several orders of magnitude. Such evidence was confirmed from both flow and stepwise tests. Flow curves highlighted significant differences in viscosity functions in terms of both magnitude and shear rate dependency. These results revealed the strong sensitivity of the rheological response of the composites to the filler type and volume fraction. Outputs of viscosity tests were modeled according to the Herschel–Bulkley model with eq 3:

$$\tau =\tau_{0}+K\cdot {\mathrm{\gamma ̇}}^{n}$$ 3

where τ and τ_0_ are the shear stress and the yield stress, respectively, γ̇ is the shear rate, *K* is the consistency index (correlated to the viscosity of the material), and *n* is a dimensionless exponent, which describes the flow behavior. A decrease in *n* values produces a more homogeneous extrusion velocity flow, ensuring a higher homogeneity of the extruded material and a better deposition on the plate after extrusion.^29^ The optimization of the model was carried out by minimizing the sum of squares of the differences between experimental data and modeled data. The τ_0_, *K*, and *n* values from fitting of the flow curves are reported in Table 2.

**Table 2:** Yield Stress (τ_0_), Consistency Indexes (*K*), and Flow Index (*n*) Calculated for the Planetary Centrifugal Mixed Slurries

composite	τ_0_ [Pa]	*K* [Pa·s^*n*^]	*n* [−]
resin	0.00	2.29	0.99
MCC23	0.00	319.25	0.38
MCC22-MFC_20_1	281.54	855.43	0.57
MCC29	0.00	1918.17	0.32
MCC28-MFC_20_1	410.71	8841.03	0.30
MCC28-MMT1	129.01	1190.42	0.42
MCC31	179.10	3198.55	0.40
MCC29-MFC_20_2	6995.61	11087.97	0.28
MCC29-MMT2	248.87	1159.87	0.45

By analyzing composites containing microcrystalline cellulose only, an increase in the volume fraction induced a general increase in the viscosity in the investigated shear rates. It is interesting to observe that by increasing the filler volume fraction the consistency index always increases, while the flow index was found to be almost stable in the range comprised between 0.3 and 0.4 at all three volume fractions. When considering the yield stress, a non-negligible value of τ_0_ was estimated at a volume fraction of 0.31. However, τ_0_ was found to be zero for both volume fractions of 0.23 and 0.29, despite the *G*′–*G*″ cross points being identified from oscillatory tests. This result can be explained by the observation that MCC23 and MCC29 are the materials that exhibited the lowest critical strains at which the transition from a predominantly solid-like behavior to a predominantly liquid-like behavior occurred, with strain values lower than 1% in both cases. It can be assumed that materials MCC23 and MCC29 are characterized by such low values of yield stress that they were not captured in the selected test conditions. The replacement of part of the MCC with the same volume of microfibrillated cellulose always caused a significant increase in both the yield stress and the consistency index when compared to the reference base materials with MCC only, as evident from the higher viscosity values in the flow curves. A clear trend was not found for the flow index that increased when microfibrillated cellulose was added in composites with a total volume fraction of 0.23, while decreased in the case of composites with total volume fractions of 0.29 and 0.31.

The use of montmorillonite also induced an increase in the yield stress but to a lesser degree. In both composites prepared with montmorillonite, the consistency *K* decreased and the flow index increased. Overall, these composites showed reduced viscosity and less shear-thinning behavior with respect to the reference materials containing microcrystalline cellulose at the same total volume fractions.

Stepwise tests (Figure 3) demonstrated that viscosity values at constant shear rate levels do not significantly vary over time, thus suggesting negligible thixotropic behavior. Moreover, it was proven that in subsequent test steps carried at the same shear rate level the dynamic viscosity was as previously measured. It was also demonstrated that after a change in the applied shear rate, the time needed to reach the reference viscosity was negligible with respect to the time of test acquisition for all materials, thus supporting the practical need of the 3D printing process.

**Figure 3:**
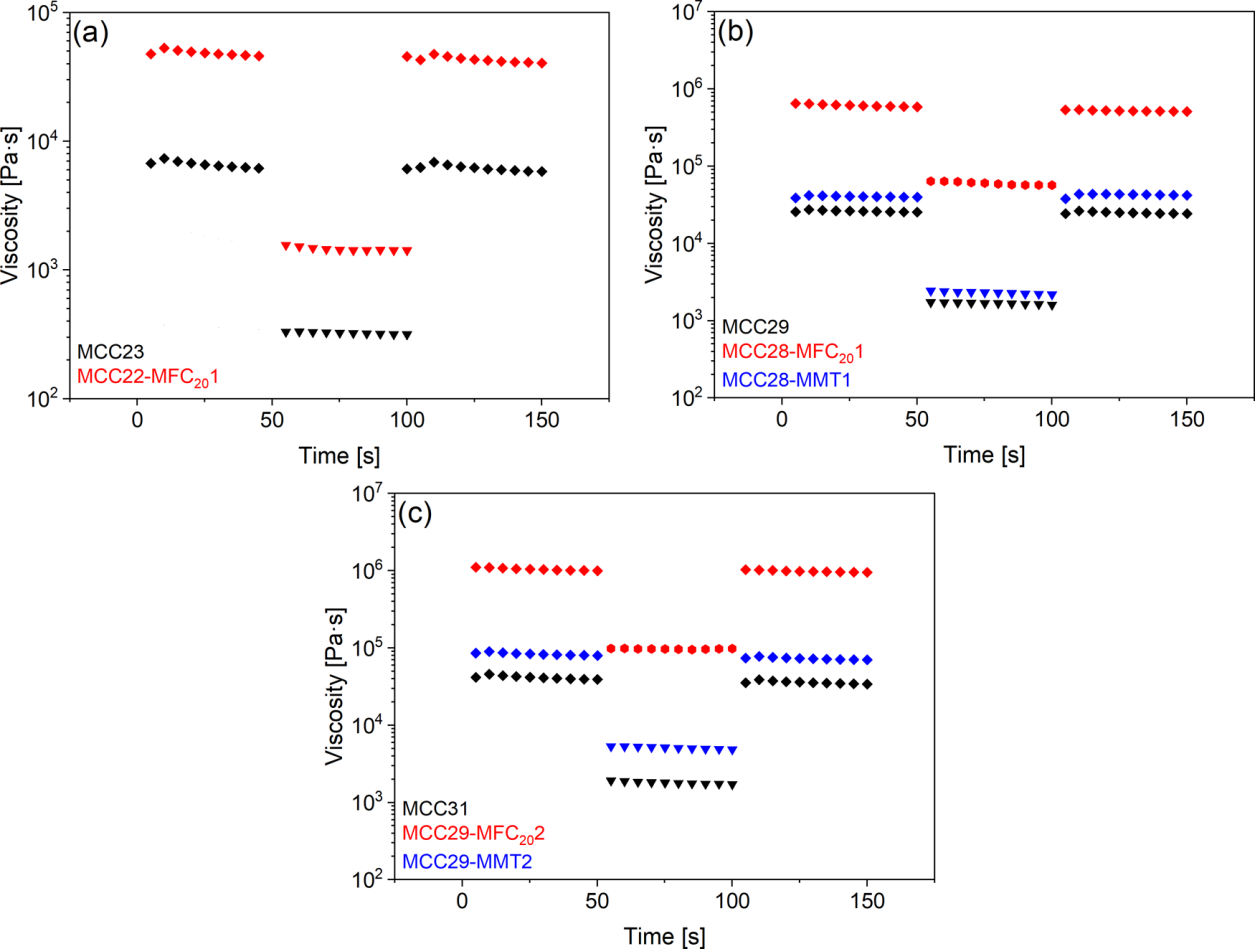
Stepwise test of the composite slurries mixed by planetary centrifugal mixing: (a) pastes with 23 vol % of filler, (b) pastes with 29 vol % of filler, and (c) pastes with 31 vol % of filler (different symbol shape indicates different shear rate value: rhombus 0.01 s^–1^, hexagon 0.1 s^–1^, and triangle 1 s^–1^).

### Curing of Resins and Composite Slurries

The FTIR spectra of NC-514S, Jeffamine ED 900, and their stoichiometric mixture (curable resin with E/AH = 1) before curing are shown in Figure S4 in the Supporting Information. In the spectrum of the curable resin, a broad band centered at 3440 cm^–1^, characteristic of the vibration of O–H bonds, was visible. This band, present also in the NC-514S epoxidized cardanol spectrum, may be associated with opened epoxy rings, as suggested by Jaillet et al.^9^ In the same region, two narrower bands at 3365 and 3297 cm^–1^ were also present, characteristic of the spectrum of Jeffamine ED 900, associated with the asymmetric and symmetric N–H stretching in primary amines. In the 3100–2700 cm^–1^ region, the bands of C–H bonds stretching appeared: the prominent absorption bands at 2927 and 2854 cm^–1^ of the epoxidized cardanol spectrum, characteristic of methylene C–H asymmetric and symmetric stretching, respectively, were clearly visible in the spectrum of the curable resin, while the complex absorption band with maximum absorbance at 2872 cm^–1^ in the spectrum of the amine hardener appeared only as a shoulder in the curable resin spectrum. The vibration of carbon–carbon bonds in the aromatic ring of the epoxidized cardanol at 1602–1584 cm^–1^ was also clearly visible in the curable resin spectrum. In this region, at 1650–1580 cm^–1^, a N–H bending vibration characteristic of primary amines but not present for secondary or tertiary amines was also visible. The characteristic band of the epoxide group appeared at 910 cm^–1^. The ATR-FTIR spectra of the uncured composite slurries (planetary centrifugally mixed) with MCC filler showed the main bands detected for the uncured resin plus the main characteristic bands of cellulose. The latter appear above 3100 cm^–1^ for hydroxyls and in the skeletal vibrations’ region of 1200–1000 cm^–1^, where an increased intensity of the band at 1040 cm^–1^ was observed, due to the C–O–C stretching in the pyranose ring. When MFC was used as filler, some differences in the profile of the characteristic peaks of cellulose were observed with respect to MCC: two separate peaks appeared in the skeletal vibrations region, namely, at 1057 and 1035 cm^–1^, and at the higher volume fractions of MFC, the hydroxyl band clearly showed a sharp signal at 3345 cm^–1^, characteristic of the O–H bond stretching vibrations of hydrogen-bonded hydroxyl groups.^30^ The band at 910 cm^–1^ characteristic of the epoxide group was visible and relatively well resolved for all of the slurries. The ATR-FTIR spectra collected for composite slurries with *V*_MCC_ = 0.23 and *V*_MCC_ = 0.29 are reported as an example in Figure S5 in the Supporting Information, where a dotted line indicates the signal of the epoxy ring at 910 cm^–1^. Thin resin films coated on silicon wafers and thick (ca. 3 mm) samples in silicon molds were cured using the time–temperature cycle described in the Experimental Section, which consists of four consecutive isothermal steps, i.e., 16 h at 40 °C, 2 h at 80 °C, 2 h at 120 °C, and 2 h at 140 °C. The curing of the resin was followed by FTIR in transmission mode and ATR mode, for the thin films and thick samples, respectively, collecting the spectra before curing and after each isothermal step of the curing cycle. For both thinfilms and thick samples, a decrease in the intensity of the band at 910 cm^–1^ was followed to monitor the conversion of the epoxy groups: the band intensity decreased with curing time and became nearly undetectable by the end of the curing cycle. In parallel, the absorption band centered at 3440 cm^–1^ increased, as hydroxyl groups were created upon the opening of the epoxide rings, and the two characteristic bands of N–H bonds in primary amines decreased. The degree of conversion of the epoxide was calculated from the decrease in the absorption band centered at 910 cm^–1^ using eq 2. The conversion was quantitative at the end of the curing cycle. For thick resin samples, the curing degree at the end of the curing cycle was similar on both sides of the specimens, the band at 910 cm^–1^ characteristic of epoxy groups being not detectable confirming full cure. The normalized ATR-FTIR spectra of the resin at the beginning and end of each curing cycle are reported in Figure 4.

**Figure 4:**
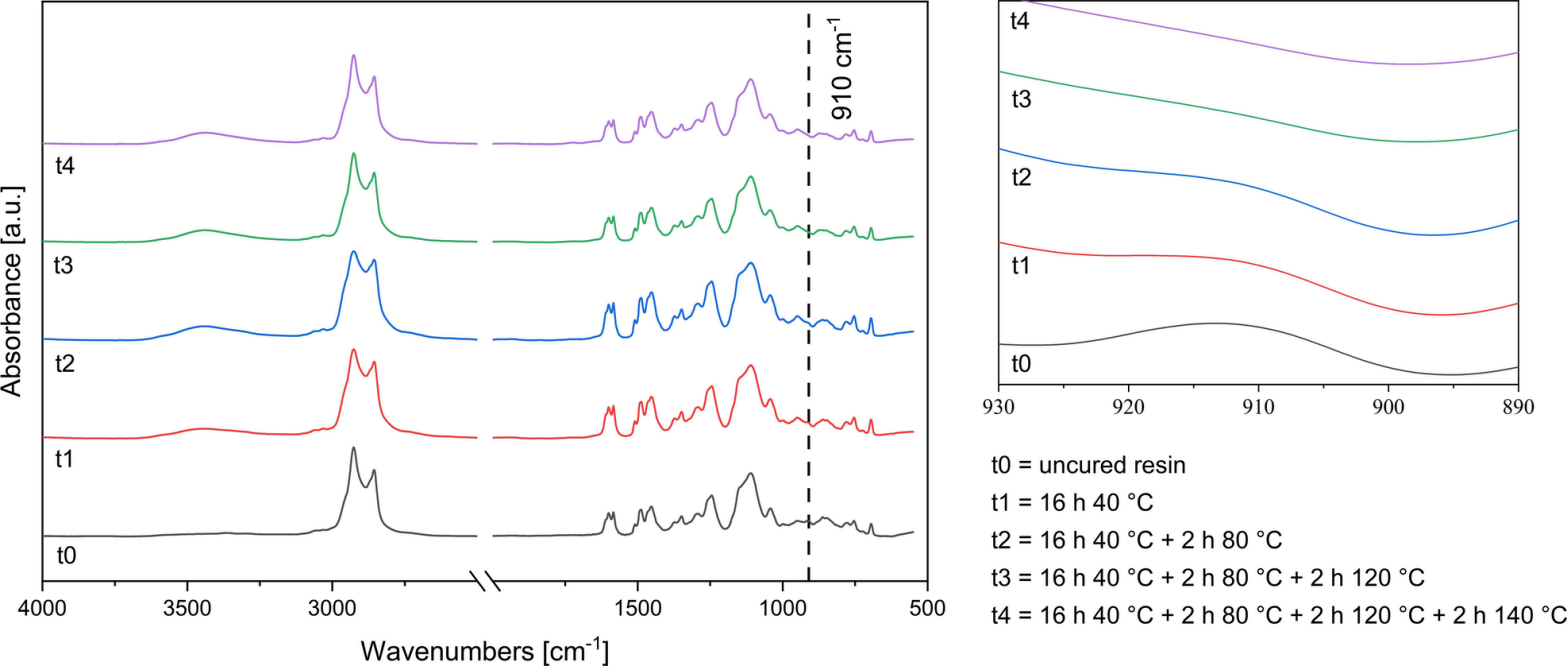
Normalized ATR-FTIR spectra of the curable resin (left) with magnification of the epoxy signal at 910 cm^–1^ (right).

Despite the complete conversion of the epoxide functionalities, the insoluble contents of nonfilled resin after immersion for 24 h in acetone and in toluene were found to be 74% and 87% by weight, respectively. Indeed, the NC-514S resin, although theoretically difunctional, contains oligomers with lower functionality; a similar epoxy/amine system was found by Jaillet et al.^9^ to have gel contents as low as 80%, possibly due to the presence of chains with opened but not reacted epoxy groups. The DSC analysis performed on the cured resin indicated a glass transition close to −32 °C, while the *T*_g_ of pristine NC-514S was −48 °C. The TGA analysis showed one thermal decomposition step, with a *T*_max_ of about 405 °C and a residual weight at 800 °C of less than 2% (see Figure S6 in the Supporting Information).

Composite slurries (also called pastes) prepared mixing the epoxy, hardener, and fillers by hand or by planetary centrifugal mixing as detailed in the Experimental Section were cured with the same four-step cycle as the resin. For each composite, the conversion of the epoxide ring after each isothermal step of the curing cycle was calculated from the ATR-FTIR spectra.

At the end of the curing cycle, quantitative conversion (>98%) was observed for all MCC composites, both mixed by hand and by planetary centrifugal mixing; the ATR-FTIR spectra are reported in Figures 5 and 6. The conversion obtained for the hand mixed MCC29 composite after the intermediate curing steps was higher than for the resin; indeed, the hydroxyl groups present on the cellulose chains, as well as in the moisture absorbed on the cellulose fibers, may catalyze the epoxy/amine reaction,^31^ thus increasing the reaction rate.

**Figure 5:**
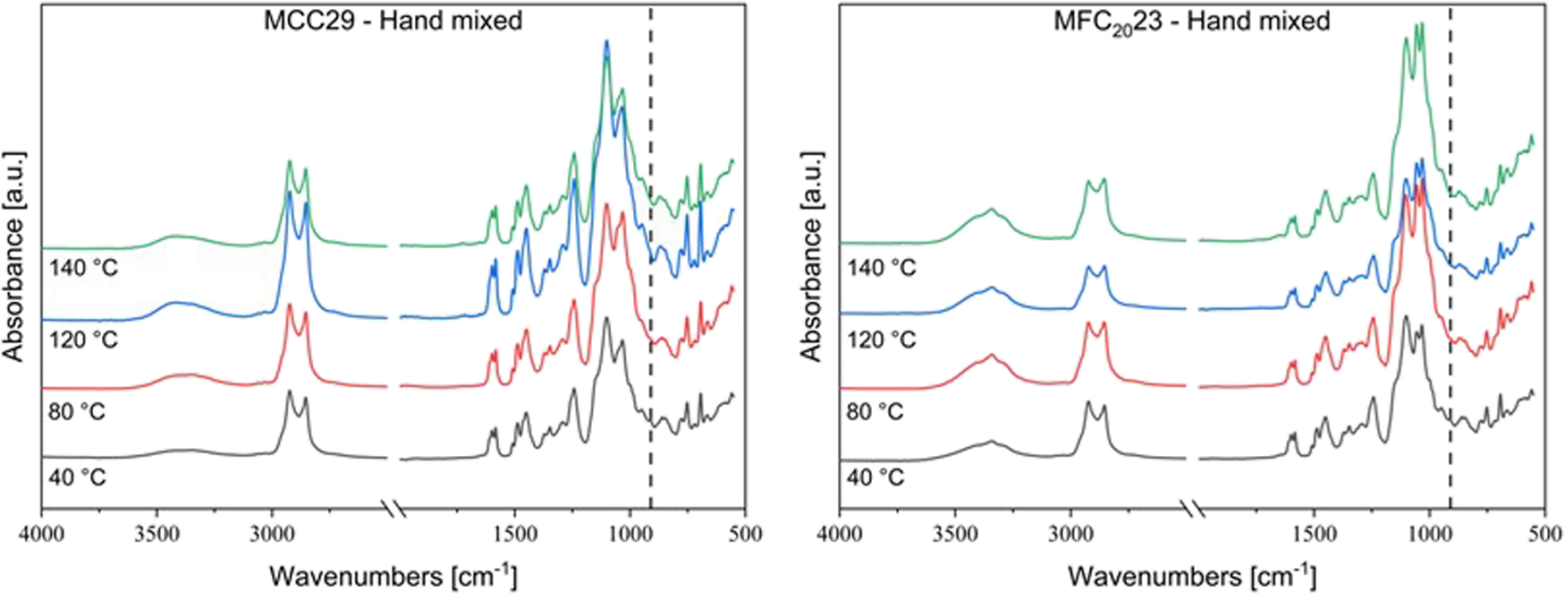
ATR-FTIR spectra of the hand-mixed composites with *V*_MCC_ = 0.29 (left) and *V*_MFC_ = 0.23 (right) after each step of the curing cycle; the dotted line at 910 cm^–1^ indicates the epoxy ring signal.

**Figure 6:**
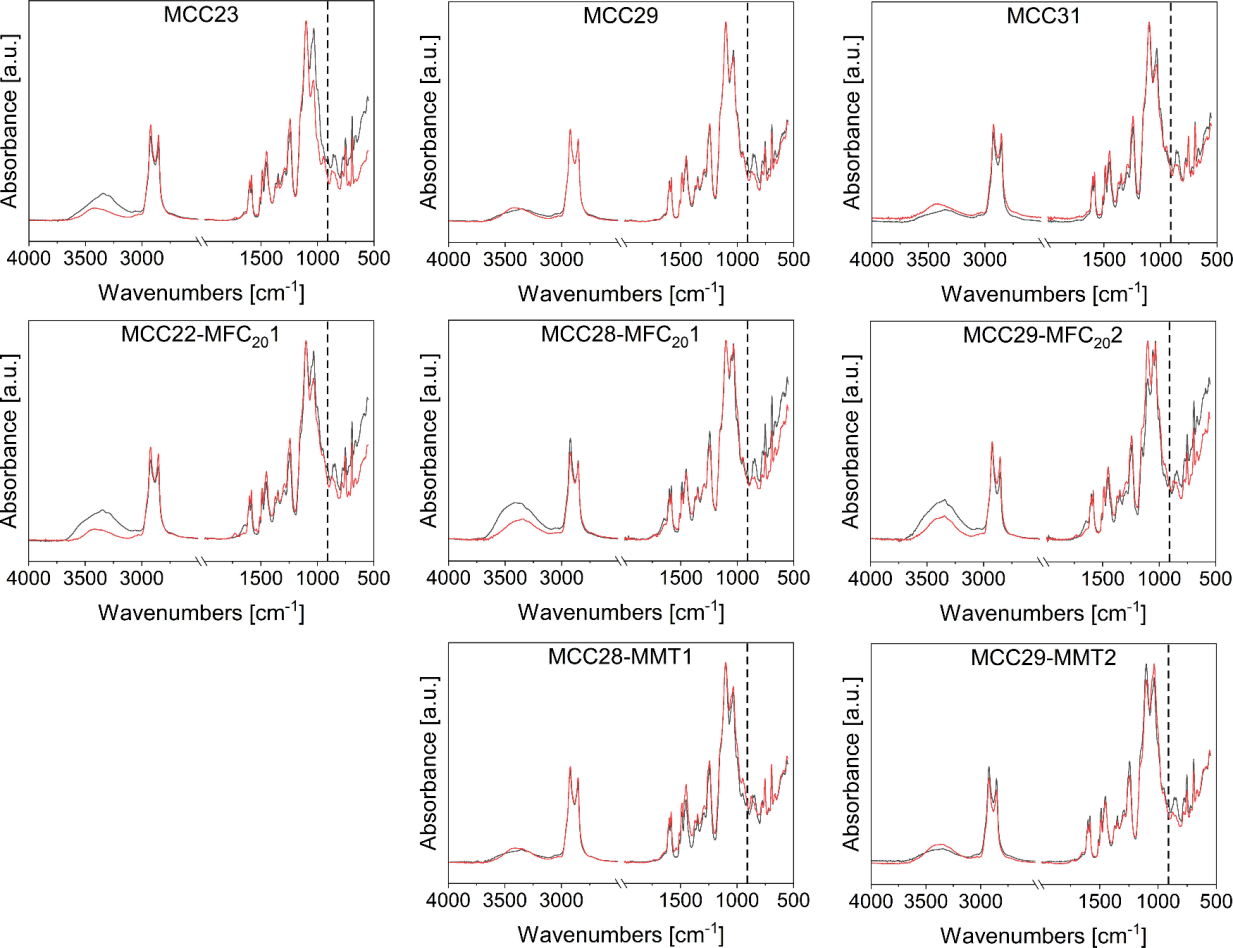
Normalized ATR-FTIR spectra of planetary centrifugally mixed composites before (black) and at the end of (red) the curing cycle; the dotted line at 910 cm^–1^ indicates the epoxy ring signal.

The final conversion of the hand mixed composites with MFC was also generally quantitative, although for the MFC_11_23 composite, which is the one with the highest water content before curing, the epoxide ring signal was still detected at the end of the curing cycle, resulting however in an overall 98% conversion.^32^

The curing of hybrid composites proceeded similarly to that of the MCC composites, generally achieving quantitative conversion of the epoxide groups. The trend in conversion for all planetary centrifugally mixed composites is shown in Figure 7: despite the reaction is almost complete for each sample, when a large amount of cellulosic filler is added (i.e., MCC31 and MCC29-MFC_20_2), the reaction rate is lower due to hindrance effects.

**Figure 7:**
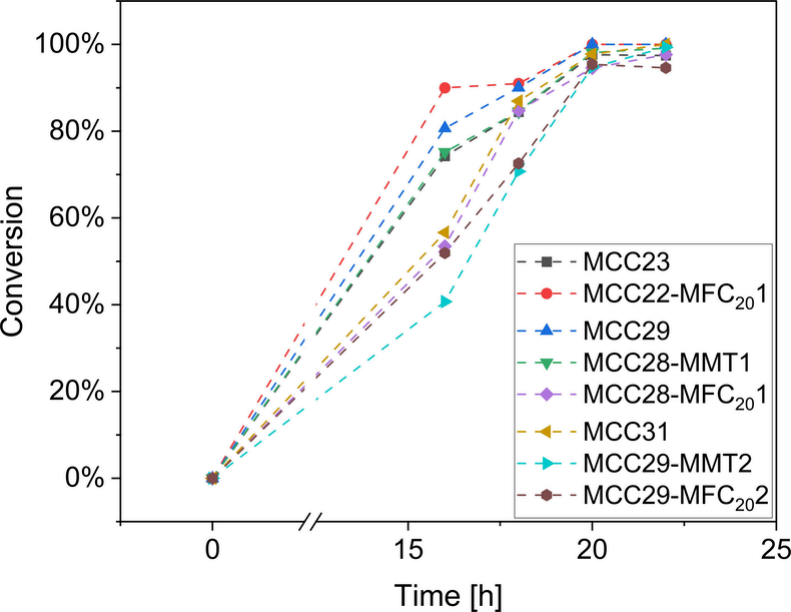
Conversion versus time for planetary centrifugally mixed samples.

The values of the insoluble content in acetone and toluene and of water uptake measured for all composites are reported in Table 3. The insoluble contents of the cured hand-mixed MCC composites measured after immersion in acetone for 24 h were all in the 87–90% range, with no significant differences with respect to the filler content. Also, the insoluble content of the composites with respect to that of the nonfilled resin increased more than was expected based solely on the amounts of insoluble fibers added. This may be due to the combination of the catalytic effect of the cellulose hydroxyls that may take part in the curing reaction and of secondary forces acting between fibers and polymer chains, e.g., hydrogen bonds. Compared to hand mixed composites prepared with MCC, those prepared with equal volume fractions of MFC had lower insoluble content: while small amounts of water, having hydroxyl groups, usually act as catalysts for the epoxy/amine reaction, large amounts of water, as those added together with MFC, may hinder the reaction.^32^ The water from MFC may even solubilize some components of the water-soluble amine hardener and thereby slow the reaction by removing them from contact with the epoxy molecules. In general, planetary centrifugal mixing slightly increased the obtained insoluble fractions of composites in acetone with MCC, to 91–92%, possibly owing to a more intimate mixing of the fibers and resin, allowing a larger resin/fiber interfacial area. When small volumes of MFC replaced an equal volume of MCC in hybrid composites, no detrimental effect on the insoluble fraction was detected. When the same volumes of MCC were replaced by equal volumes of clay, a slight increase in the insoluble content (by weight) was detected, which may be ascribed to the higher density of clay with respect to cellulose. In general, insoluble fractions in toluene were higher than those in acetone.

**Table 3:** Insoluble Content and Water Uptake

composite	insoluble content in acetone [%]	insoluble content in toluene [%]	water uptake [%]
resin	74 ± 0	87 ± 0	31
hand mixing			
MCC7	87 ± 0	92 ± 0	40
MCC23	90 ± 0	95 ± 1	38
MCC29	89 ± 2	92 ± 0	36
MFC_20_7	84 ± 1		44
MFC_20_23	77 ± 0		46
MFC_11_7	80 ± 0		52
MFC_11_23	78 ± 0		47
			
planetary centrifugal mixing			
MCC23	90 ± 0	92 ± 0	25
MCC22-MFC_20_1	85 ± 3	88 ± 0	28
MCC29	91	93 ± 0	32
MCC28-MFC_20_1	90	92 ± 0	27
MCC28-MMT1	91	97 ± 0	25
MCC31	92	95 ± 1	28
MCC29-MFC_20_2	93	94 ± 1	29
MCC29-MMT2	95	94 ± 0	24

Water uptake was higher for samples mixed by hand mixing, suggesting planetary centrifugal mixing made composites with a lower number of voids. Furthermore, water uptake increased when MFC was used to replace part of the MCC; for the same vol % of filler, composites with MMT showed a water uptake slightly lower than the one of composites with the same volume fraction of MFC.

### Morphology and Mechanical Properties of Cured Composites

Photos of the cured resin and composites are shown in Supporting Information. While cured resin samples (Figure S7a) were brownish but transparent, the composites were not transparent, regardless of the type of filler and mixing method used.

Hand-mixed composites with the MCC filler (Figure S7b) had a light caramel color and were homogeneous through the thickness; at the higher MCC contents, the high viscosity of the pastes resulted in a rough surface. Shrinkage upon curing was negligible. Composites with *V*_MFC_ = 0.07 looked relatively homogeneous, while with *V*_MFC_ = 0.23, poorer mixing between fibers and matrix was evident, particularly with the more concentrated suspension, MFC_20_, and the two sides of the specimens had a different appearance (Figure S7c,d). Also, due to the evaporation of water during curing, the composites with a higher amount of MFC showed very high shrinkage, proportionally to the initial water content; the shrinkage of MFC_11_23 was so marked that it was not possible to obtain proper specimens for mechanical testing, as shrinking inside the mold caused strain and damage.

Composites mixed by planetary centrifugal mixer are shown in Figure S8: they were not transparent and became darker with a higher vol % of filler. The addition of MMT resulted in a significant color change. In general, they appeared homogeneous through thickness. Shrinkage was negligible also for composites containing MFC due to the small amount added.

FESEM observation of freeze-fractured cross sections of samples cured in molds (see the Supporting Information, FESEM) containing 29 vol% of MCC showed that macroscopic voids were present in both hand mixed and planetary centrifugally mixed composites due to the high viscosity of the materials, making it difficult to spread them in the molds; however, with planetary centrifugal mixing, a more homogeneous dispersion of the filler was obtained, as shown in the images with higher magnification. Increasing the amount of MCC to 31 vol% caused the appearance of more macroscopic and microscopic voids, while the MCC dispersion in the matrix remained good. Hybrid materials with both MFC and MMT also showed macroscopic spherical voids at all concentrations, possibly due to entrapped air and smaller irregular voids that can be ascribed to the high viscosity making spreading in the mold difficult. An overall good dispersion of the fillers was reached, although for MFC hybrids, few aggregates and filler free area were visible. At higher magnification, clay platelets can also be identified.

Stress–strain curves of hand-mixed (including resin without fillers) and centrifugal mixed composites are reported in Figures S9 and S10, respectively. The stress–strain curves obtained from tensile tests for the unfilled resin exhibit nonlinearity in the low strain region, which may be associated with a rearrangement at the molecular level. Then, at strains above 0.3 mm/mm, the stress–strain curve increases almost linearly until rupture, at 0.87 mm/mm (87%) deformation, with and ultimate tensile strength of 0.22 ± 0.03 MPa. The Young’s modulus was 0.35 ± 0.05 MPa (initial region).

The stress–strain curves of MCC composites, independently of the mixing method, were linear and showed a rather brittle rupture. Increasing the MCC content increased the modulus and tensile strength while decreasing the elongation at break. The moduli of MCC23 and MCC29 composites obtained by hand mixing and planetary centrifugal mixing were similar; with the highest MCC content (MCC31, prepared with planetary centrifugal mixer), a 16-fold increase of the modulus was obtained, with a reduction of elongation at break of 80% with respect to the pristine resin.

Tensile test curves for hand-mixed composites with exclusively MFC are reported in Figure S9. Nevertheless, when comparing these data with the other here reported, it should be remembered that the final dimensions of the specimens were different and some internal stresses may have developed due to shrinkage during curing in the mold. At the lowest *V*_MFC_, the stress–strain curves were linear with brittle rupture, similar to that of the materials containing MCC, although with the less concentrated MFC, some nonlinearity appeared in the initial part of the curve. By the addition of the higher amount of MFC, the onset of nonlinear stress–strain behavior, indicating plastic deformation involving structural rearrangement of the material, is observed already at very low strain. Larger standard deviations were obtained for the results, particularly for tensile strength, which may indicate the presence of defects in the prepared specimens, e.g., voids, as also shown by the FESEM micrographs. With comparable filler volume fractions, the moduli obtained with MFC were 1 order of magnitude larger than the corresponding ones with MCC, confirming the higher reinforcing effect of microfibrillated cellulose.

The composites with hybrid MCC-MFC filler prepared with the planetary centrifugal mixer showed an increase of the mechanical properties, for both modulus and tensile strength, with a decrease in the maximum strain values, compared to samples with same volume of only MCC. The curve with 1 vol % of MFC (for MCC28-MFC_20_1) showed a linear behavior, while by adding a higher amount of MFC, i.e., 2 vol % for MCC29-MFC_20_2, a change of the slope was observed (as seen for sample MFC_20_23). The obtained moduli increased about 21-fold and 51-fold with respect to the pristine resin and of 1.3-fold and 3-fold with respect to the homologues containing only MCC. Standard deviations were in general low, showing good homogeneity between samples.

The composites with hybrid MCC-MMT filler mixed with the planetary centrifugal mixer showed an increase of the mechanical properties for both modulus and tensile strength, too, with respect to homologues containing only MCC, although not as much as for hybrids with MFC. However, in this case, a decrease in the maximum strain values was not observed. Standard deviations were, in general, lower than the ones for hybrid MCC-MFC samples, showing a better homogeneity of the samples.

Eventually, the sample MCC29-MFC_20_2 had the best mechanical properties among the hybrid samples, with a Young’s modulus of 17.84 ± 1.28 MPa and a tensile strength of 1.71 ± 0.18 MPa. A summary of the mechanical properties for each sample is given in Table 4.

**Table 4:** Tensile Properties

composite	Young’s modulus *E* [MPa]^a^	Ultimate tensile strength σ [MPa]	ε_max_ [mm/mm]
resin	0.35 ± 0.05	0.22 ± 0.03	0.87 ± 0.12
hand mixed			
MCC7	1.13 ± 0.03	0.66 ± 0.04	0.65 ± 0.02
MCC23	3.76 ± 0.08	1.04 ± 0.13	0.30 ± 0.05
MCC29	6.51 ± 0.17	1.78 ± 0.06	0.30 ± 0.02
MFC_20_7	14.83 ± 1.68	1.97 ± 0.43	0.16 ± 0.02
MFC_20_23	70.56 ± 4.81	2.01 ± 0.31	0.08 ± 0.01
MFC_11_7	13.15 ± 1.45	2.10 ± 0.96	0.24 ± 0.10
MFC_11_23	/	/	/
			
planetary centrifugal mixing			
MCC23	3.59 ± 0.19	0.76 ± 0.15	0.23 ± 0.03
MCC22-MFC_20_1	5.34 ± 0.50	1.31 ± 0.16	0.28 ± 0.01
MCC29	5.38 ± 0.26	1.33 ± 0.08	0.25 ± 0.02
MCC28-MFC_20_1	7.29 ± 0.48	1.04 ± 0.15	0.15 ± 0.02
MCC28-MMT1	6.21 ± 0.20	1.51 ± 0.18	0.25 ± 0.03
MCC31	5.61 ± 0.28	0.90 ± 0.09	0.17 ± 0.02
MCC29-MFC_20_2	17.84 ± 1.28	1.71 ± 0.18	0.14 ± 0.01
MCC29-MMT2	9.39 ± 0.48	1.60 ± 0.20	0.18 ± 0.02

a Calculated from the slope of the linear part.

### 3D Printing by Liquid Deposition Modeling

The most promising slurries were selected for preliminary 3D printing trials. The slurries with only MFC filler were discarded because of their very high shrinkage upon curing, which would undermine the final shape fidelity. On the other hand, slurries with the higher volume fractions of MCC-MFC hybrid filler proved impossible to extrude, as they did not present a solid to liquid transition in the desired strain range. Thus, MCC22-MFC_20_1, MCC31, and MCC29-MMT2 underwent printing, as they presented the highest storage moduli at rest and proper solid to liquid transitions.

MCC31 and MCC29-MMT2 while being easily extruded presented poor shape fidelity, as the paste gradually collapsed under the weight of the superposed layers (Figure S11). Furthermore, the high amount of filler tended to clog the extruder nozzle reducing the extrusion speed during the printing process. On the other hand, printing of MCC22-MFC_20_1 resulted in good shape fidelity with a good shape retention after the curing process. The 1 vol % of MFC inside the formulation enhanced the printability of the paste, giving good shear-thinning behavior without shrinkage during cross-linking in the oven. An example of a 3D printed cylinder is reported in Figure 8.

**Figure 8:**
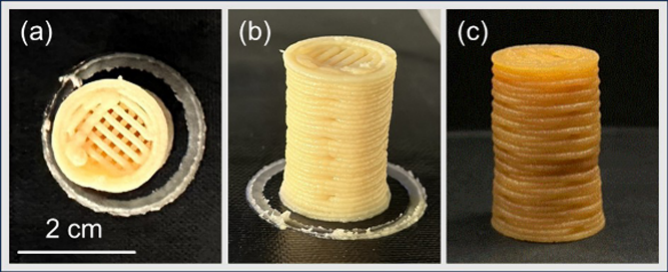
Printed slurry MCC22-MFC_20_1 (a, b) before and (c) after curing.

## Conclusions

In this work, microcrystalline cellulose (MCC), microfibrillated cellulose (MFC), and clay (MMT) were investigated as rheology modifiers and mechanical properties enhancers in biobased epoxy composites for liquid deposition modeling 3D printing. Planetary centrifugal mixing allowed for the improvement of the dispersion of fillers at high loadings, with respect to hand mixing. Addition of MCC led to a shear-thinning behavior of the slurries, with a solid-like behavior to liquid-like behavior transition in the region of 0.5–5 strain %. The stepwise tests showed negligible thixotropy, enabling rapid structural recovery that is crucial for maintaining shape fidelity and ensuring reliable layer stacking in liquid deposition modeling (LDM) 3D printing. Replacing MCC with MFC in the slurries enhanced shear-thinning behavior; however, despite MFC providing a significantly stronger reinforcing effect, pronounced shrinkage highlighted the need to remove the water introduced with MFC prior to curing. Replacing small amounts of MCC with equal volumes of clay did not substantially alter the rheological behavior of the slurries with respect to MCC alone; however, a slight increase of the tensile properties of the cured slurries was observed. Substitution of small amounts of MCC with equal amounts of MFC instead led to a radical change in rheological behavior, enhancing the shear-thinning behavior with an abrupt increase in viscosity with respect to the MCC composites, while solid-like behavior was maintained almost in the entire test region at higher filler loadings. Rapid viscosity recovery was retained. These composites showed the highest moduli and strengths (the highest registered for MCC29-MFC_20_2), confirming the excellent reinforcing effect of MFC. Tensile moduli of composites with 29 vol % and 31 vol % filler increased by 36% and 218% by replacing 1 vol % and 2 vol % of MCC with MFC, respectively. Finally, the MCC22-MFC_20_1 slurry was used for preliminary 3D printing trials by LDM showing proper extrusion and good shape fidelity before and after curing.

## Supplementary Material



## Notes

Chemical structures of NC-514S epoxy resin and ED 900 cross-linker, conversions calculated from FTIR spectra for the resins E/AH = 1:1 and E/AH = 0.8, optical micrographs of cross sections of centrifugally mixed MCC29 composites cured with different cross-linking cycles, FTIR spectra of NC-514S, Jeffamine ED 900, and their stoichiometric mixture, ATR-FTIR spectra of the composite slurries with VMCC = 0.23 and VMFC = 0.29 and of hybrid composites slurries with VMMT = 0.02 and VMFC = 0.02, DSC graphs for samples MCC31, MCC29-MMT2, and MCC29-MFC_20_2 and TGA for cross-linked sample with E/AH ratio = 1, pictures of cured hand mixed samples and planetary centrifugal mixed composites, stress–strain curves of bare resin, hand-mixed MFC composites and planetary centrifugal mixed composites, 3D printed MCC31 paste showing collapsing of the structure (PDF); FESEM images of cross sections of hand-mixed MCC29 and all planetary centrifugally mixed composites (PDF)
