# Supplementary material for: Liquid Deposition Modeling of Biobased Epoxy Composites: Natural Fillers as Rheology Modifiers and Reinforcements
Source: ACS Omega. 2026 Feb 6;11(6):9996–10007. doi: 10.1021/acsomega.5c10820 (PMC12917825; doi:10.1021/acsomega.5c10820)
Supplement: Supplementary file 1 [file ao5c10820_si_001.pdf]

Liquid Deposition Modeling of biobased epoxy composites: natural fillers as rheology modifiers and reinforcements

Edoardo Albertini[1], Christos Fragkogiannis[1], Lucia Tsantilis[1], Rossella Arrigo[2], Alessandra Vitale[1], Roberta Bongiovanni[1], Sara Dalle Vacche[1]*

[1]Politecnico di Torino, Corso Duca degli Abruzzi 24, 10129, Turin, Italy

[2] Politecnico di Torino, Viale Teresa Michel, 15121 Alessandria, Italy

A)

B)

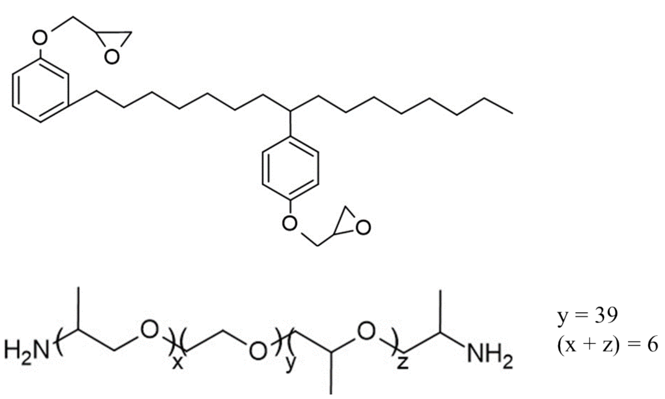

Figure S1. Chemical structures of (a) NC-514S epoxy resin, (b) Jeffamine ED 900 polyetheramine hardener.

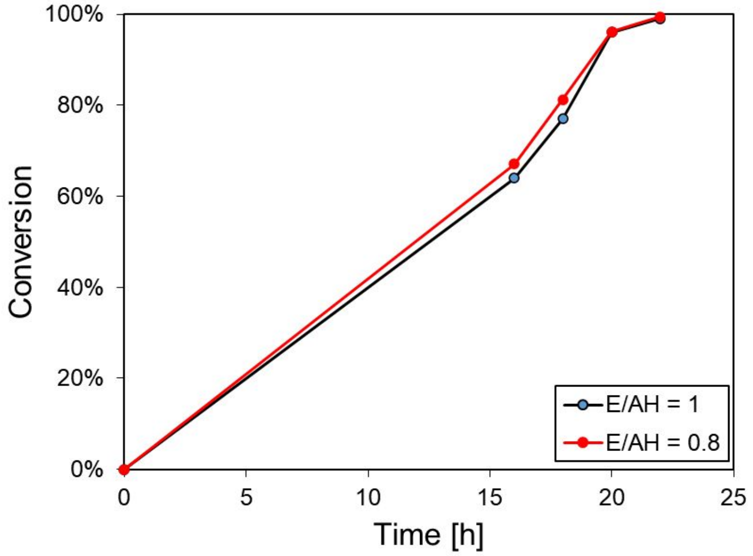

Figure S2. Conversions calculated from FTIR spectra for the resins E/AH = 1:1 and E/AH = 0.8

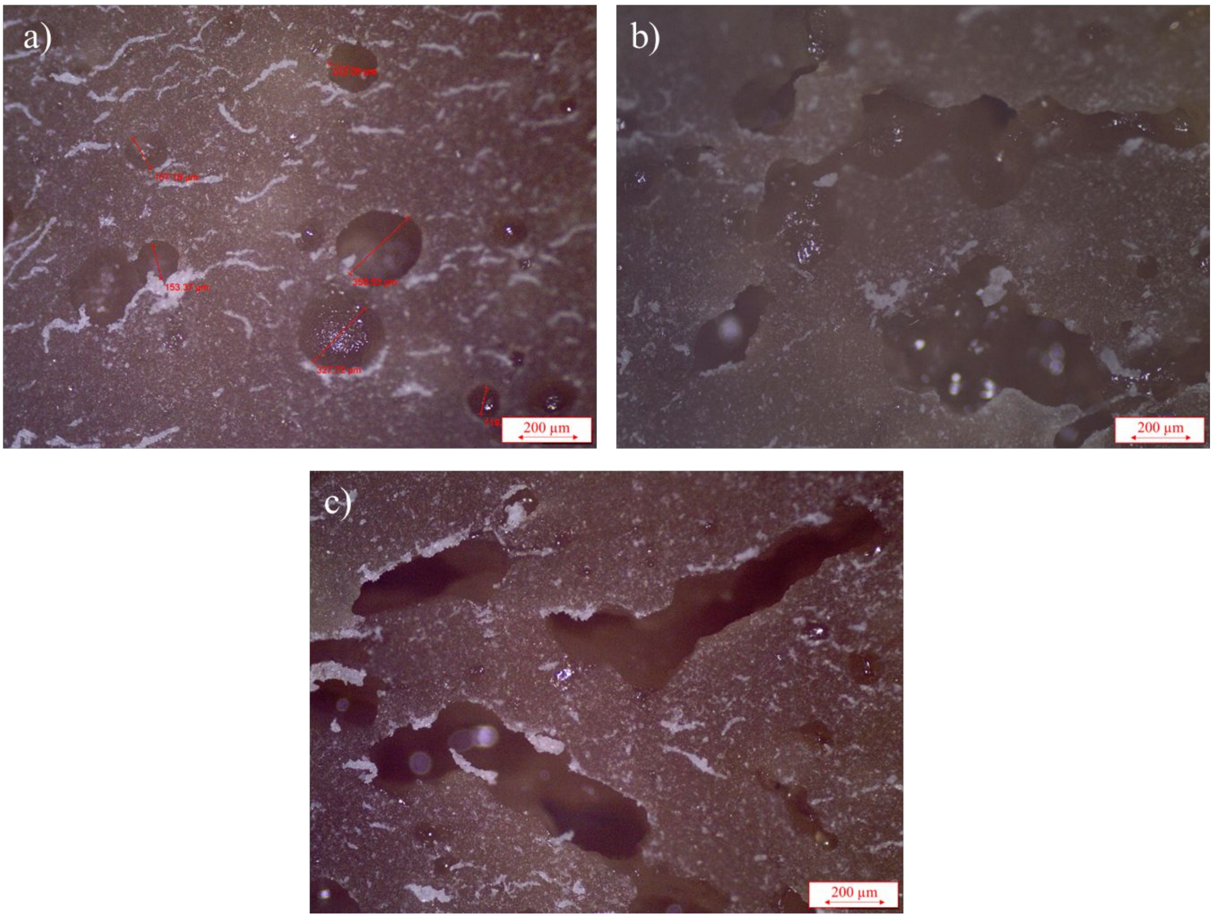

Figure S3. Optical micrographs of cross-sections of centrifugally mixed MCC29 composites cured with different cross-linking cycles: (a) complete curing cycle, (b) curing cycle without the 16 h step at 40 °C and (c) curing cycle without the 16 h step at 40 °C and the 2 h step at 80 °C.

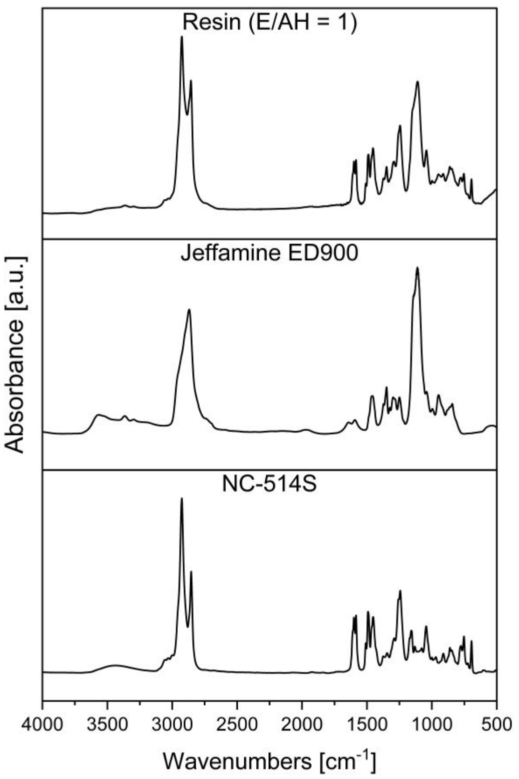

Figure S4. FTIR spectra of NC-514S, Jeffamine ED 900 and their stoichiometric mixture (resin).

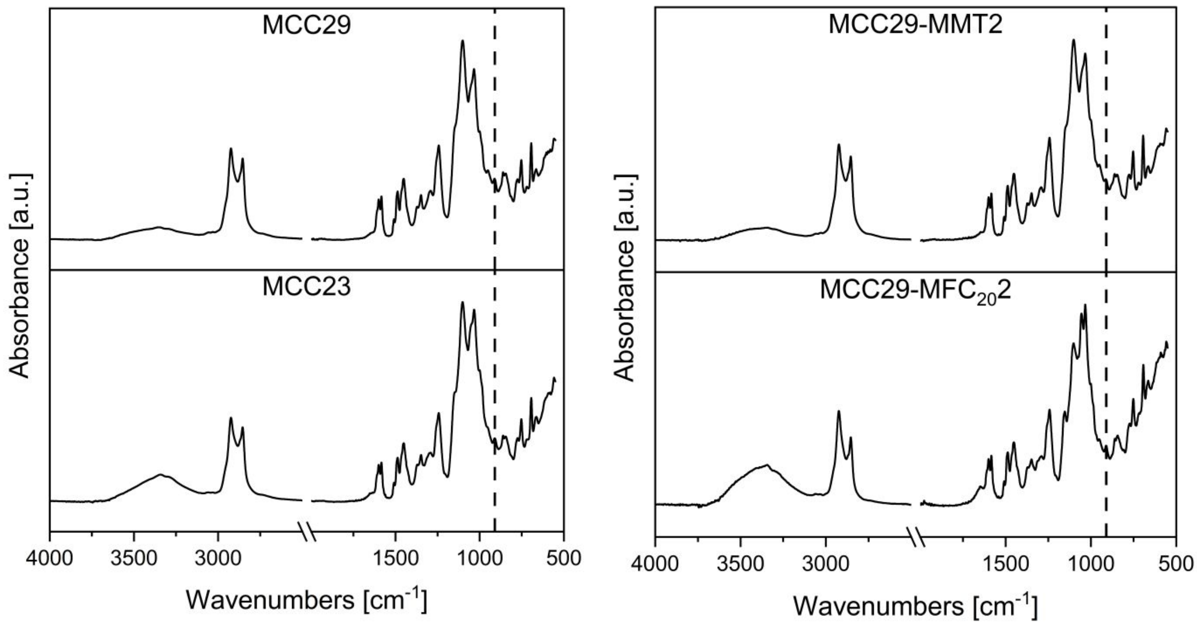

Figure S5. Normalized ATR-FTIR spectra of the composite slurries with VMCC = 0.23 and VMFC = 0.29 (left) and of hybrid composites slurries with VMMT = 0.02 and VMFC = 0.02 (right) (planetary centrifugally mixed samples); dotted line at 910 cm-1 indicates the epoxy ring signal.

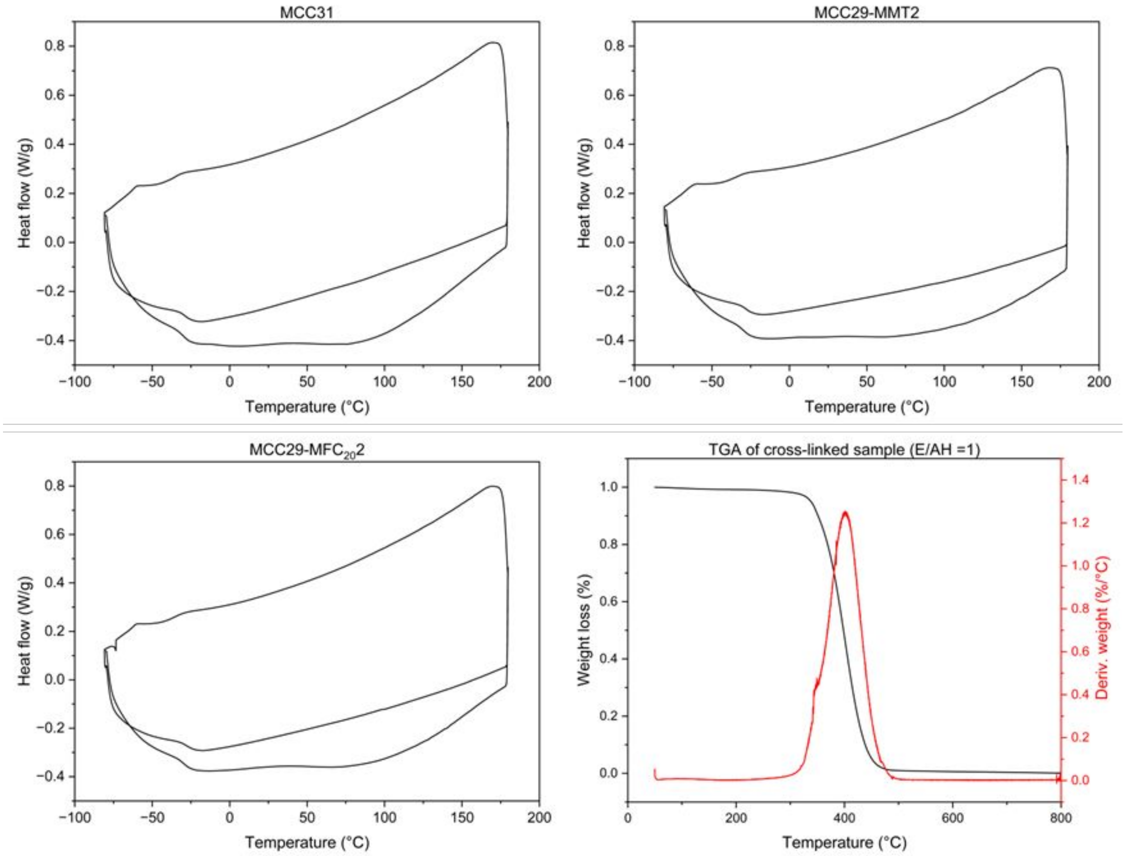

Figure S6. DSC graphs for samples MCC31, MCC29-MMT2 and MCC29-MFC_{20}2 and TGA for cross-linked sample with E/AH ratio = 1.

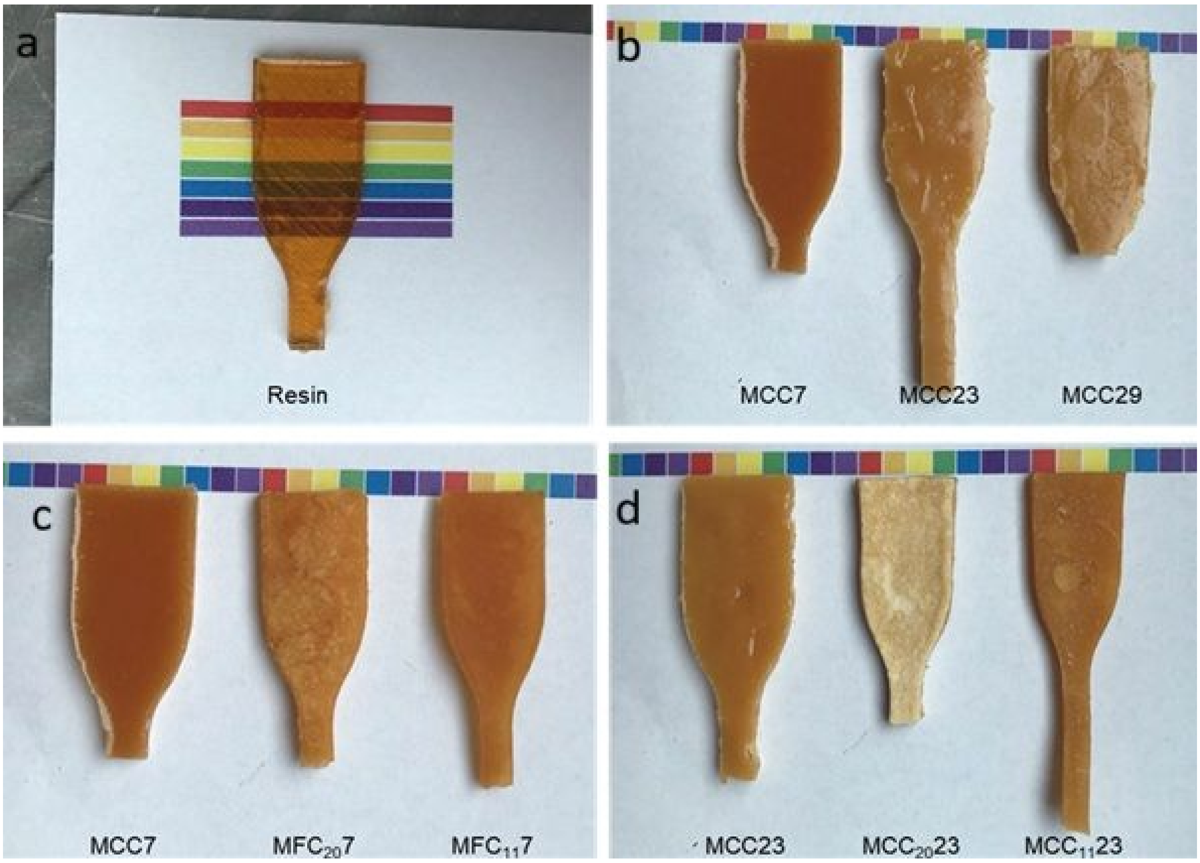

Figure S7. Photos of cured hand mixed samples: (a) cured resin, (b) composites with only MCC filler, (c) composites with φ_{MCC} and φ_{MFC} = 0.07 and (d) composites with φ_{MCC} and φ_{MFC} = 0.23.

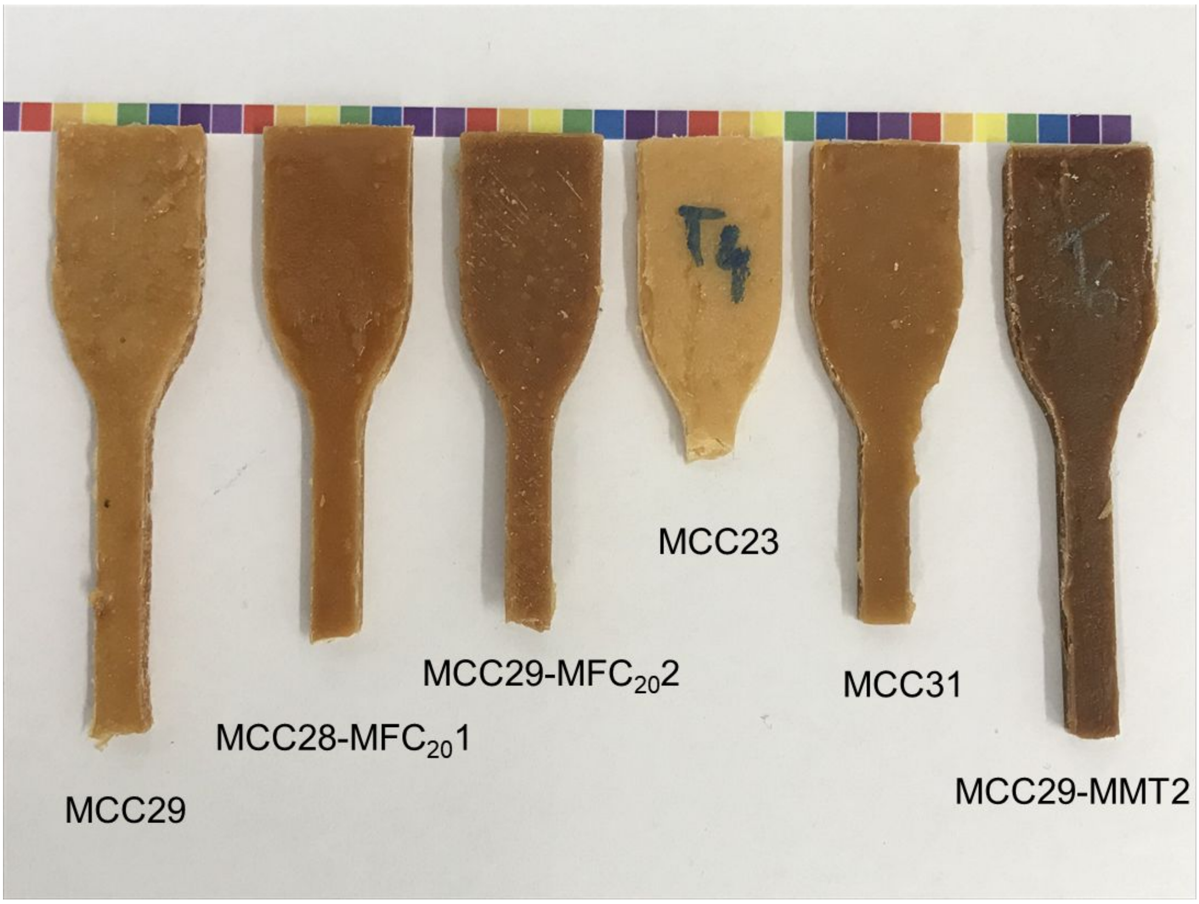

Figure S8. Planetary centrifugal mixed composites.

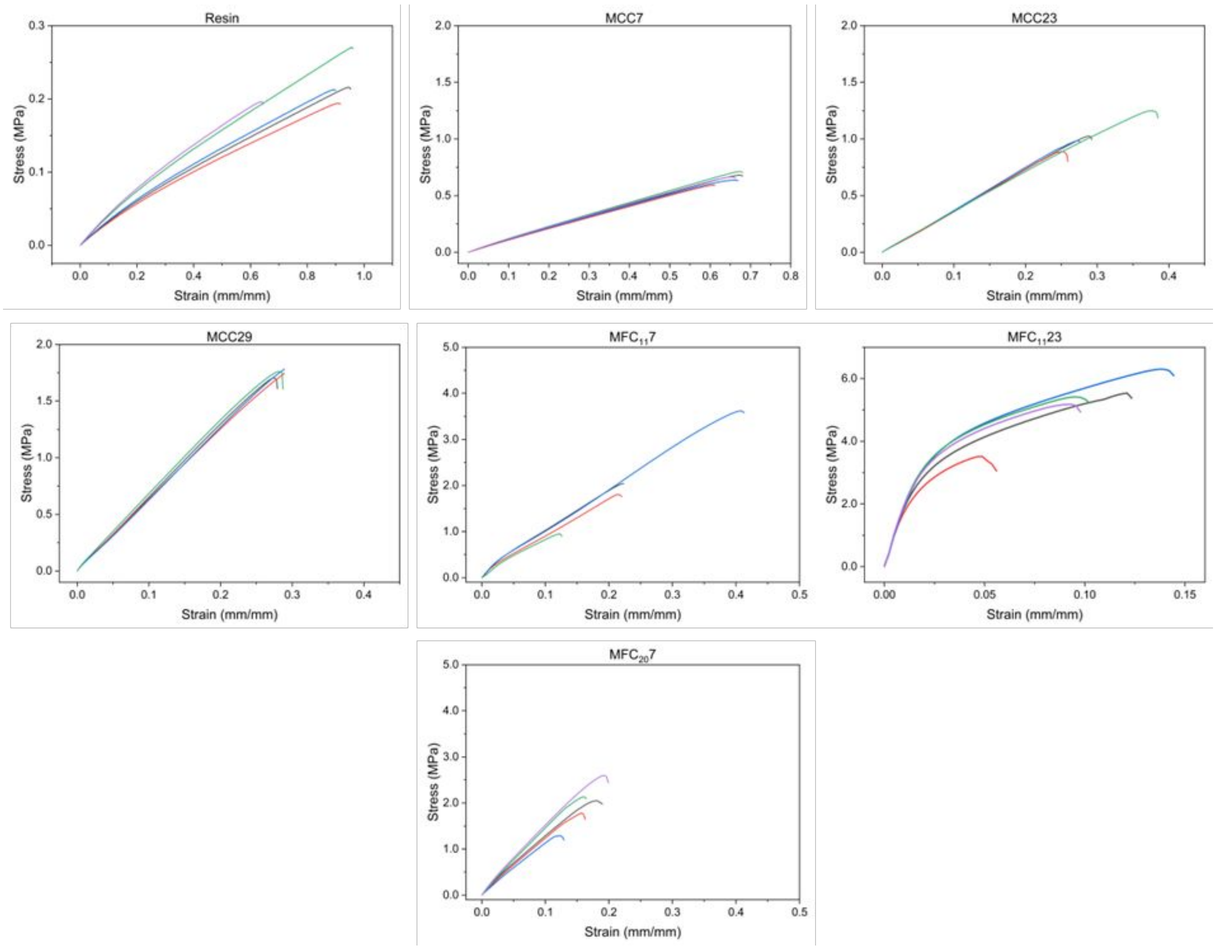

Figure S9. Stress-strain curves of bare resin and hand mixed MFC composites.

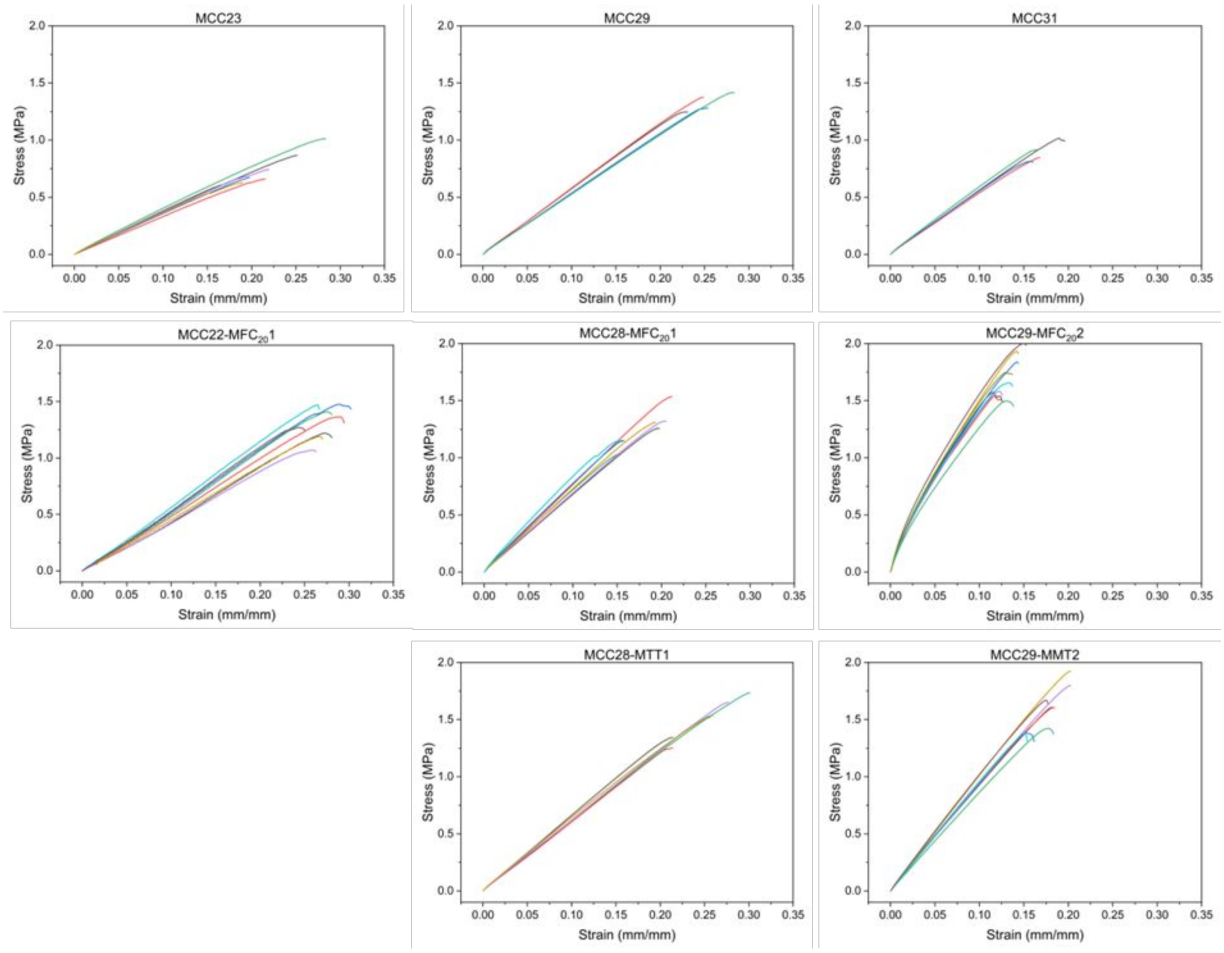

Figure S10. Stress-strain curves of planetary centrifugal mixed composites.

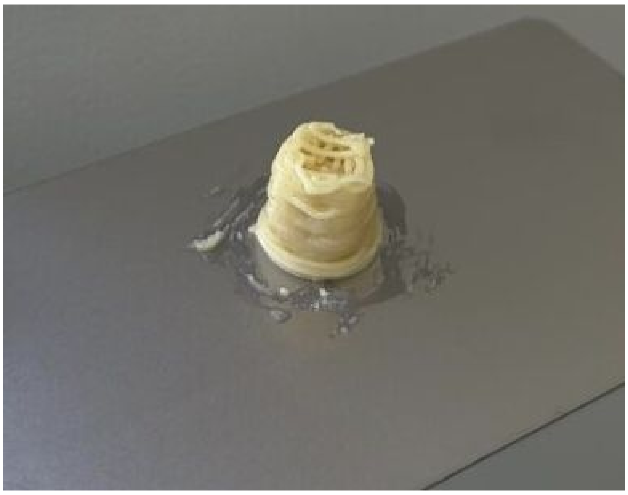

Figure S11. 3D printed MCC31 paste showing collapsing of the structure.

1. Selection of the resin formulation.

To select the most suitable ratio of epoxy resin to amine hardener, two epoxy to amine hydrogen (E/AH) ratios were explored in this work: stoichiometric (E/AH = 1) and slight excess of amine hydrogen (E/AH = 0.8). While the curable resin with excess hardener showed slightly higher reactivity particularly during the curing steps at the lower temperatures, as shown by the conversion values calculated from FTIR analysis (Figure S2), the insoluble content after immersion for 24 h in acetone was found to be 74±0.8 % and 70±1.6 % by weight for E/AH = 1 and E/AH = 0.8, respectively. The lower gel content obtained with the latter could possibly be attributed to either an increased fraction of soluble oligomers or to the presence of excess unreacted hardener. The Young's modulus of the cured resin with excess hardener (0.26 ± 0.01 MPa) was slightly lower than that of the stoichiometric resin, and the elongation at break (1.05 ± 0.13 mm/mm) was slightly higher, owing to the very flexible polyethylene glycol chain of Jeffamine ED900 hardener. The ultimate tensile strength was similar (0.19 ± 0.03 MPa). Finally, no significant difference in T_g or thermal stability was detected between the cured resins with the different E/AH ratios. Thus, as despite a moderately higher reactivity, the final properties were slightly diminished adding excess hardener, the stoichiometric formulation was selected for the preparation of composite materials.
